# Morphological and transcriptional evaluation of multiple facial cutaneous hyperpigmented spots

**DOI:** 10.1002/ski2.96

**Published:** 2022-02-04

**Authors:** T. Hakozaki, B. Jarrold, W. Zhao, T. Laughlin, D. Whittenbarger, E. A. Jewell‐Motz, R. E. Boissy

**Affiliations:** ^1^ The Procter & Gamble Company Mason Business Center Mason Ohio USA; ^2^ Department of Dermatology College of Medicine University of Cincinnati Cincinnati Ohio USA

## Abstract

**Background:**

Morphological characteristics of major facial hyperpigmented spots have been well documented. However, detailed alterations of respective transcriptional profile for each spot and in‐depth comparisons across multiple spot types have not been reported.

**Objectives:**

To comprehensively assess and compare multiple facial hyperpigmented spot types at the morphological and molecular levels by utilising transcriptional expression profiling with correlation to quantified histological features.

**Methods:**

Multiple types of facial spot biopsies were collected from Chinese women and compared to additional biopsies taken from adjacent healthy skin. The types of spots included Solar Lentigos with both elongated dermal‐epidermal junction (DEJ) (SL[E]) and flat DEJ (SL[F]), Seborrhoeic Keratosis (SK), Melasma, Freckles, Post‐inflammatory hyperpigmentation of resolving acne (PIH[A]) and other stimuli (PIH[O]). Combined histomorphometry, immunohistology, and transcriptome analysis for suprabasal‐epidermis, basal‐epidermis, and dermal compartments dissected by Laser Capture Microdissection (LCM) were conducted and compared across different spot types.

**Results:**

Each spot type was confirmed to have the unique histological pathology already documented elsewhere. Most of the spot types except Melasma and PIH (A) revealed similar melanocyte density to adjacent skin. All spots exhibited increased melanin synthesis, melanosome transportation, as well as enhanced melanocyte dendricity, however, each spot revealed a distinct transcriptome regulation pattern in pigmentation pathways. Upregulation of pigmentation genes was also observed in the dermis of SL(F), SL(E), SK and PIH(O), associated with significant modulation of DEJ related genes in basal‐epidermis and/or dermal compartments, suggesting potential melanocyte infiltration into the dermis due to impaired DEJ quality. Beyond upregulated pigmentation, for most spots, gene expression in the suprabasal‐epidermis regulating keratinisation was significantly upregulated in conjunction with thickened stratum corneum. Furthermore, downregulation of tight junction related genes represented by claudin‐1 was observed in majority of spot types, suggesting compromised barrier function could be a similarity across spots. Additionally, Cyclin‐Dependent Kinase Inhibitor 2A (CDKN2A) was upregulated in all types of spots, indicating involvement of cell senescence as a common theme.

**Conclusion:**

This comprehensive and comparative study based on the histological and transcriptional analysis of three skin compartments provided unique insights into specific causations as well as differences and similarities across multiple hyperpigmented spot types.

## Graphical abstract

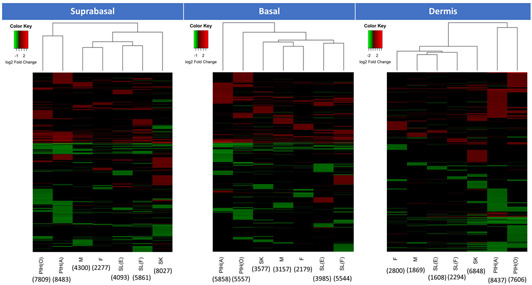

1

**What's already known about this topic?**

- There are multiple types of cutaneous hyperpigmented spots which exhibit distinct morphologic profiles. However, the underlying molecular mechanisms have not been fully elucidated yet.

**What does this study add?**

- Detailed transcriptional profiling and histological comparisons across (1) multiple hyperpigmented spots and (2) three cutaneous compartments (suprabasal‐epidermis, basal‐epidermis and dermal) independently using Laser Capture Microdissection technique with a relatively large basesize in a comprehensive manner.
- Interesting biological similarities across multiple hyperpigmented spots beyond upregulated melanin synthesis such as thicker stratum corneum due to upregulated keratinisation, impaired dermal‐epidermal junction and skin barrier function, and accumulation of senescent cells compared to adjacent normal skin.

**What is the translational message?**

- Understanding the molecular basis of hyperpigmented spots will help develop intervention strategies to improve the appearance of hyperpigmented spots uniquely customised to each spot type or targeting the biological similarities to effectively improve multiple spot types simultaneously.

## 2 INTRODUCTION

There are various classifications of benign hyperpigmented spots occurring on human skin with not fully understood aetiologies. Spots may form in response to multiple factors such as UV exposure and/or hormonal changes.^1^, ^2^ The most common spots are Melasma, Solar Lentigo (SL), Freckles, pigmented Seborrhoeic Keratosis (SK) and post‐inflammatory hyperpigmentation (PIH), which can be clinically and histologically differentiated.^3^, ^4^, ^5^, ^6^, ^7^ Epidermal Melasma^8^ is defined as a light to dark brown or slate, irregular hypermelanosis of the face and forearms occurring primarily in women. Histologically epidermal melanosis demonstrates melanin deposition mainly in the basal and suprabasal‐epidermis layers. Melanocytes are normal to slightly increased in number and are enlarged with prominent dendrites. SL^9^ develops with age and is induced by solar or ultraviolet light exposure. Histologically lesions exhibit hyperplasia of the epidermis with variable melanocyte number in the basal‐epidermis layer. The dermal‐epidermal junction (DEJ) can be flattened or pendulous. Freckles^10^ are congenital and generally confined to sun‐exposed body areas. Histologically there is an increase in basal melanin with a slight increase in melanocyte density and variable rete ridge elongation. SK^11^ can be brown, black or tan but distinguishable with a waxy, scaly, elevated appearance. Histologically they demonstrate hyperkeratosis and papillomatosis. PIH^12^ results from a variety of external or immune assaults on the skin including acne scaring and may or may not be temporary. Because all these hyperpigmented spots possess distinctive histological features, we presumed different transcriptional and pathophysiological mechanisms underlie their development.

Understanding differences in their aetiologies is essential for the development of intervention strategies for distinct spots. We used Transcriptional Expression Profiling or Bioinformatics Cluster Analysis, a widely used tool for understanding possible molecular mechanisms underlying complex disease processes^13^ including many skin diseases^14^, ^15^, ^16^ as well as melanoma.^17^, ^18^ We combined this tool with histological analysis to characterise distinctive pathology and underlying molecular biology of multiple hyperpigmented spots.

## 3 MATERIALS AND METHODS

### 3.1 Subject population

The study was approved by the RCRC Independent Review Board. All subjects provided written informed consent before study procedures. 77 Chinese females, aged 18–70, in good general health with at least one spot of interest (SL, SK, PIH, light symptom melasma, freckles) as diagnosed by the study dermatologist were enroled at Biometrix in San Francisco, California. Exclusion criteria included; scarring; cosmetic facial procedures; hormone replacement, anti‐inflammatories or immunosuppressants therapies; hydroquinone or retinoids treatments; and systemic diseases. A survey queried spot acquisition history was used to sub‐classify PIH into specifically acne‐induced PIH (PIH[A]) from other chemical/physical stimuli‐induced PIH (PIH[O]).

### 3.2 Skin biopsy samples

Two‐millimetre punch biopsies were collected from the target facial spot and adjacent non‐spot areas. If the subject possessed more than one type of spot, up to two types of spots were collected. Non‐spot biopsies were selected to be free of any visible inhomogeneous colour, such as telangiectasia, dyspigmentations, scars, or rash. Biopsies were embedded in Optimum Temperature Compound (OCT) (Sukura Finetec) before freezing over liquid nitrogen and stored at −80°C until cryostat sectioning at 10 µm. Spot classifications were confirmed by dermatopathological examinations by a dermatologist and a dermatopathologist using haematoxylin and eosin (H&E)‐stained skin sections. SL samples were also sub‐classified based on the histopathological analysis of rete ridge (elongated; SL[E], flat; SL[F]) as previously described.^19^

For transcriptome analysis, biopsies were separated into three compartments by Laser Capture Microdissection (LCM) to enrich cell biological information. Basal epidermis, supra‐basal epidermis and dermal LCM fractions were collected from ten 14 µm serial sections of each biopsy and pooled to create one sample per fraction for each subject from which total RNA was isolated. The dermis is defined as the tissue remaining following the removal of all epidermal appendages (hair follicles, sebaceous glands and sweat glands) and subcutaneous adipose tissue.

### 3.3 Histomorphometry

Histomorphometry was conducted to observe structural alterations in different spot types compared to non‐spot tissue using H&E‐stained images. Image Pro Premier software (Media Cybernetics) was used to measure epidermal thickness, rete ridge content and stratum corneum thickness in each biopsy. Viable epidermal thickness was measured by tracing lines along both the DEJ and the epidermal granular layer then calculating the average distance between these lines across the entire epidermal length. Rete ridge content was measured taking the ratio of the DEJ length to that of the granular layer. Stratum corneum thickness was measured by tracing two separate lines along both the epidermal granular layer and the top of the stratum corneum then calculating the average distance between these lines across the entire epidermal length.

### 3.4 Immunohistology of Pmel17, CDKN2A and microphthalmia transcription factor (MITF)

Immuno‐histological assessment of CDKN2A and Pmel17 were performed using an anti‐CDKN2A/p16INK4a (Abcam, ab189034) or an anti‐Pmel17 (Abcam, ab63297) antibody and DAPI counterstained using NucBlue fixed cell stain Ready Probes reagent (ThermoFisher). For comparison, fluorescent images of spot and non‐spot biopsies were captured with a Zeiss Observer.Z1 microscope (Carl Zeiss Microimaging, Germany) at equal gamma values, pixel range and exposure.

Identification/quantification of melanocytes in non‐spot and spot biopsies (*N* = 4 subjects for each spot type) was accomplished through MITF staining utilising a MITF antibody (C5/D5 monoclonal, Sigma 284M‐97) and a Histostain plus kit (ThermoFisher) with DAB as a chromogen according to manufacture recommendations. MITF positive cell nuclei in basal‐epidermis were counted across the entire length of the DEJ of each biopsy. To account for differences in DEJ undulation between spot types and non‐spot tissue, all counts were normalized to 2 mm of total DEJ length.

### 3.5 Melanin staining

Ten micrometre fresh frozen sections were fixed, washed, and stained according to the manufacturer's instructions using the Fontana Masson stain kit (Abcam, ab150669).

### 3.6 Transcriptome analysis, statistics, bioinformatics and data presentation

Transcriptome analysis was performed by using Affymetrix GeneTitan U219 array plates (Affymetrix) with the Affymetrix GeneTitan instrument and protocol provided. Following processing, chip images were converted to numeric data using the PLIER algorithm as executed in the Affymetrix Gene Chip Expression Console. R packages (R Core Team: http://www.R-project.org/) were used for statistical analyses. Differentially expressed probe sets for each spot versus non‐spot adjacent tissue were analysed using One‐way ANOVA model implemented in the lima R package. Hierarchical cluster analysis of spot gene expression data (log2 fold changes of spot vs. non‐spot adjacent tissue) was done using heatmap.2 R package. Only probe sets which are significantly changed (*p* < 0.05) in at least one spot type were included in the clustering analysis. Gene set enrichment analysis (GSEA) was done using GAGE^20^ implemented in R. Graphs of regulation of human pigmentation genes from The International Federation of Pigment Cell Societies (IFPCS: https://www.ifpcs.org/colorgenes/) were generated for each spot type using Cytoscape.^21^

Details of LCM and RNA isolation processes, histomorphometry, immunohistology, as well as mRNA target labelling, processing, and analysis are provided in Supporting Information S1.

## 4 RESULTS

### 4.1 Spot‐type characterisation based on dermatopathological examination

Collected spot tissues were categorised into seven types based on the on‐site diagnosis and dermatopathological examination by utilising the H&E histology images. The demography by spot types is summarised in Table 1.

**TABLE 1:** Demography of characterised spot types based on the dermatopathological examination

Type of spot	Code	Sample number
Solar lentigo (elongated dermal‐epidermal junction [DEJ])	SL(E)	15
Solar lentigo (flat DEJ)	SL(F)	21
Seborrhoeic Keratosis	SK	25
Melasma	M	13
Freckles	F	12
Post‐inflammatory hyperpigmentation (acne induced)	PIH(A)	12
Post‐inflammatory hyperpigmentation (other stimuli)	PIH(O)	14

### 4.2 Histological evaluation of spot types

**FIGURE 1:**
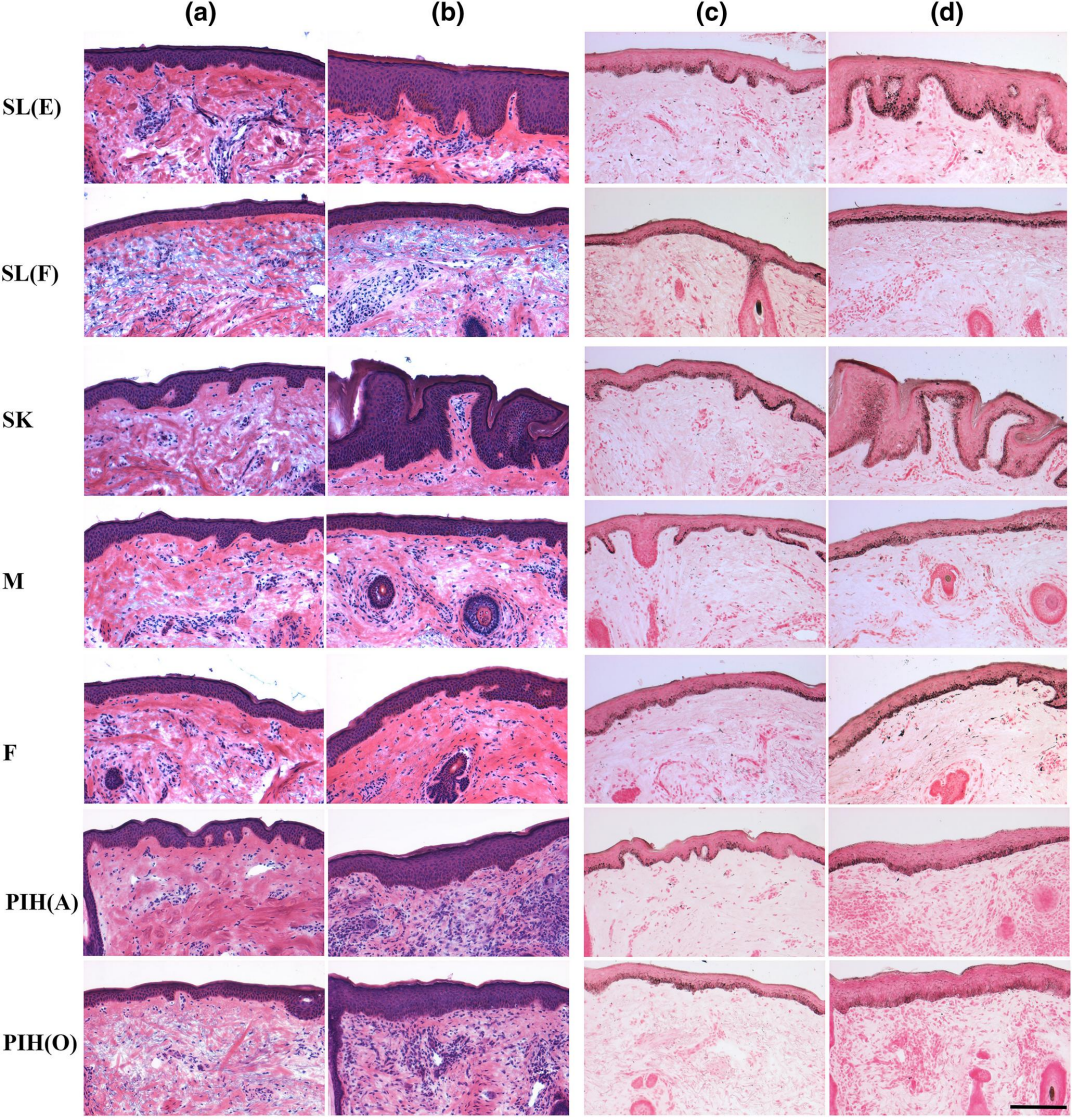
Representative images of haematoxylin & eosin (Columns a and b) and Fontana Masson (Columns c and d) stained skin biopsies of both non‐spot (Columns a and c) and seven different spot types (Columns b and d). Scale bar = 200 microns

**FIGURE 2:**
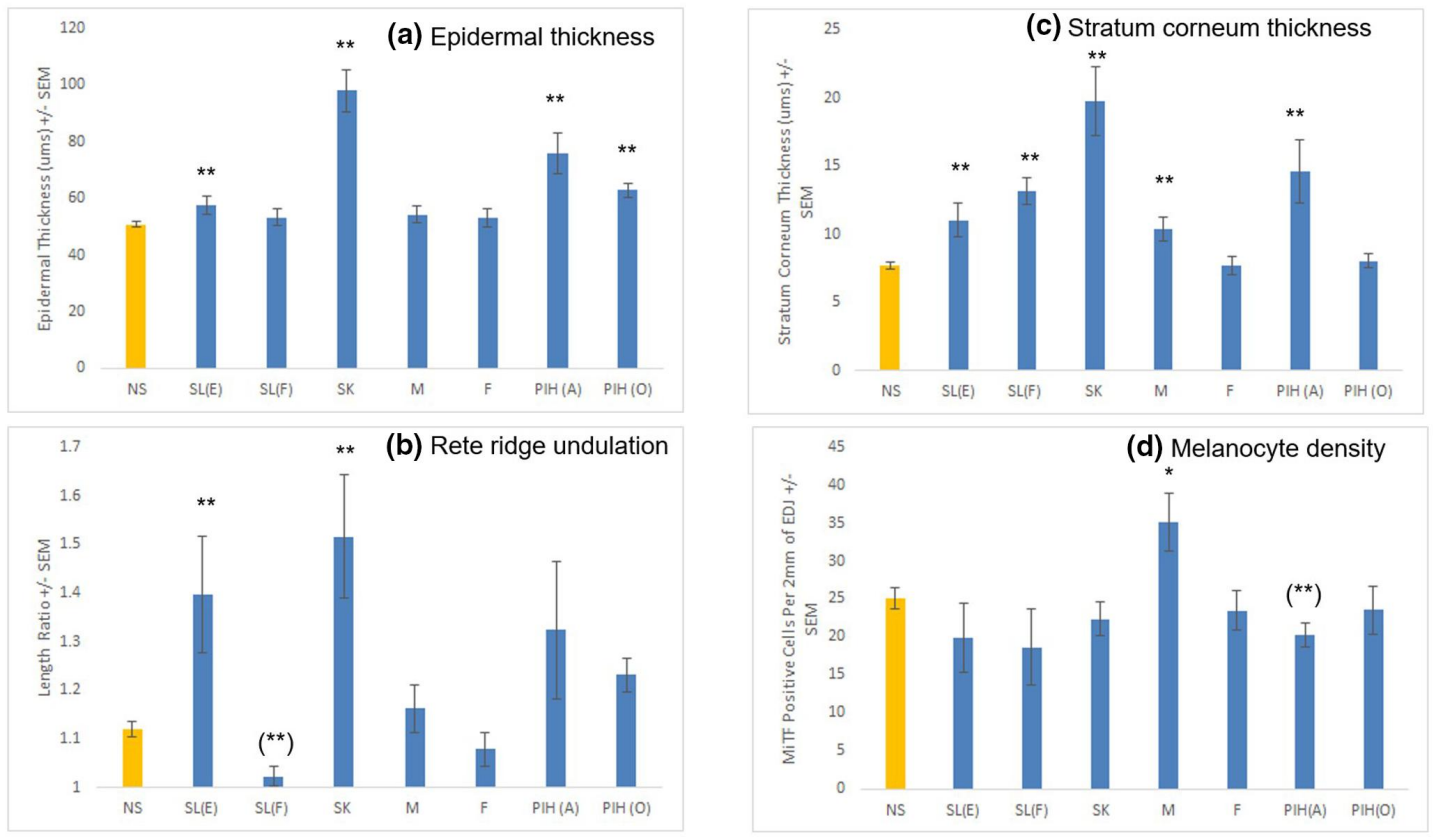
Skin feature analysis on (a) epidermal thickness (b) rete ridge undulation (c) stratum corneum thickness from H&E images and (d) melanocyte density from MITF images. Mean ± SEM. **p < 0.05, *p < 0.1 versus non‐spot (NS) by student *t*‐test. () indicates significantly different to lower direction

Histological/morphometric characterisation of the seven classified spot types compared to non‐spot skin was investigated through H&E and Fontana‐Masson staining (Figure 1). Multiple spot types: SL(E), SK, PIH(A) and PIH(O) exhibited epidermal hyperplasia which resulted in significant increases in viable epidermal thickness (Figure 2a). The epidermal hyperplasia in SL(E) and SK was accompanied by significant increases in DEJ undulation/rete ridges (Figure 2b). In contrast, SL(F), Melasma and Freckles demonstrated no epidermal thickening, while significant flattening of the DEJ was evident in SL(F). Interestingly, significant thickening of the stratum corneum was noted for the majority of spot types: SL(E), SL(F), SK, Melasma and PIH(A) (Figure 2c).

**FIGURE 3:**
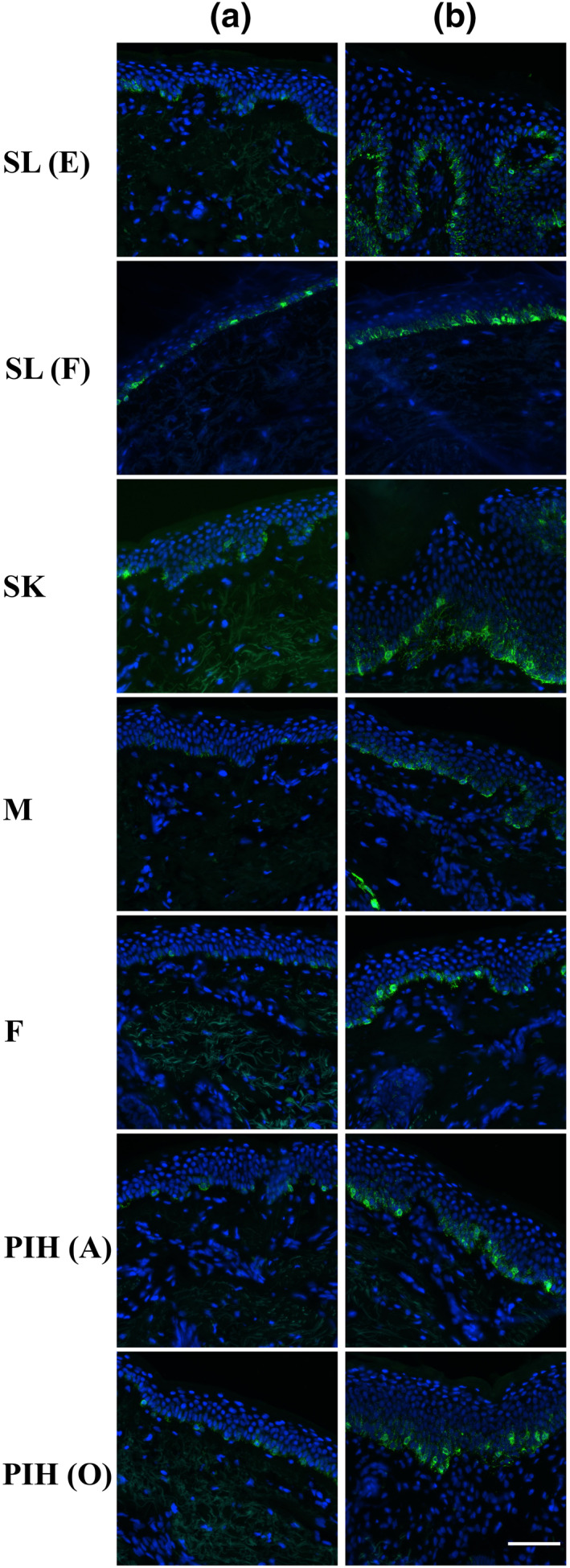
Representative images of Pmel17 immunofluorescent staining of skin biopsies from both non‐spot (Column a) and 7 different spot types (Column b). Scale bar = 100 microns

Fontana‐Masson staining revealed that qualitatively all spot types except PIH(O) have a higher melanin content than non‐spot tissue with predominate melanin localization to the basal‐epidermis layer (Figure 1). Melanocyte density measurement via MITF immunohistochemistry suggested the observed melanin content increase is due to increased melanin production and not increased number of melanocytes as only Melasma demonstrated an increase in density (Figure 2d). To further investigate melanin production, we conducted staining for Pmel17 that illustrates all spot types have a higher melanosome abundance and increased melanocyte dendricity compared to non‐spot samples (Figure 3).

### 4.3 Transcriptional evaluation of melanogenesis and DEJ integrity in spot types

**FIGURE 4:**
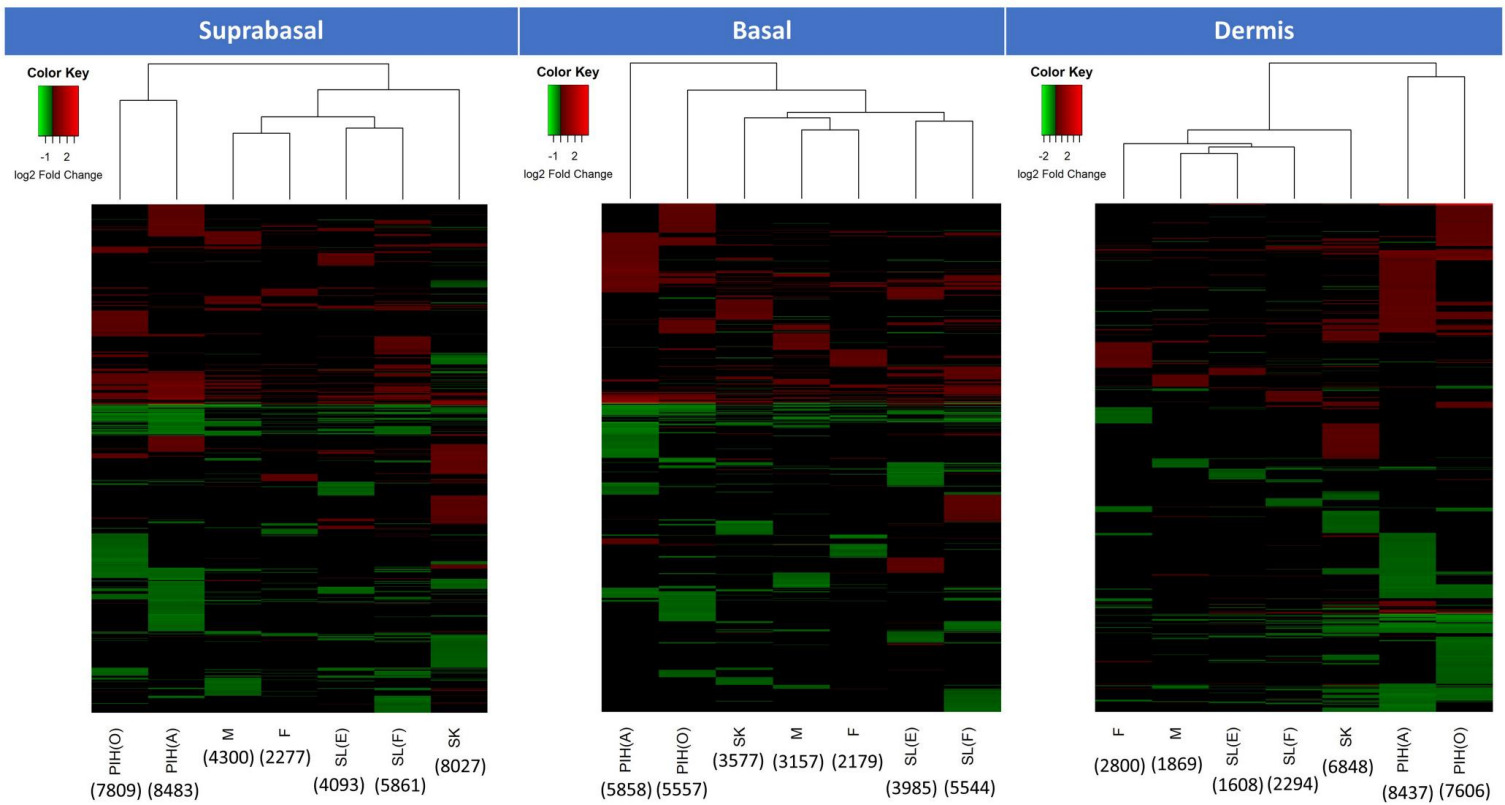
Overall gene expression patterns of seven spot types in each layer; suprabasal epidermis, basal epidermis and dermal. Cluster analysis (groupings above the heat maps) indicate the similarity of expression patterns of each spot within each skin layer. Green indicates downregulation, red indicates upregulation with colour intensity signifying magnitude of fold change. Numbers in parenthesis under spot names are significantly modulated probe set numbers versus adjacent normal skin (*p* < 0.05). Total probe set numbers of used Affymetrix GeneTitan U219 array plate is 49 293

The heatmap of significantly changed genes indicated clearly different patterns in all spot types across three skin layers (Figure 4). Hierarchical cluster analysis indicated Freckles and Melasma are more like each other, as well as SL(E) and SL(F). PIH(A) and PIH(O) are also like each other and distinctively different from other spots. We further investigated the regulation of pigmentation genes in different spots. Among 153 human pigmentation related genes from IFPCS, 145 genes were significantly regulated in the basal‐epidermis layer in at least one spot type. Cytoscape network graphs are shown for each spot type (Figure S1). SK showed a dramatically different expression pattern, with most pigmentation genes downregulated. SL(E), SL(F) and Freckles showed more similarity in the regulation of pigmentation genes. PIH(A) and PIH(O) are more like each other.

**FIGURE 5:**
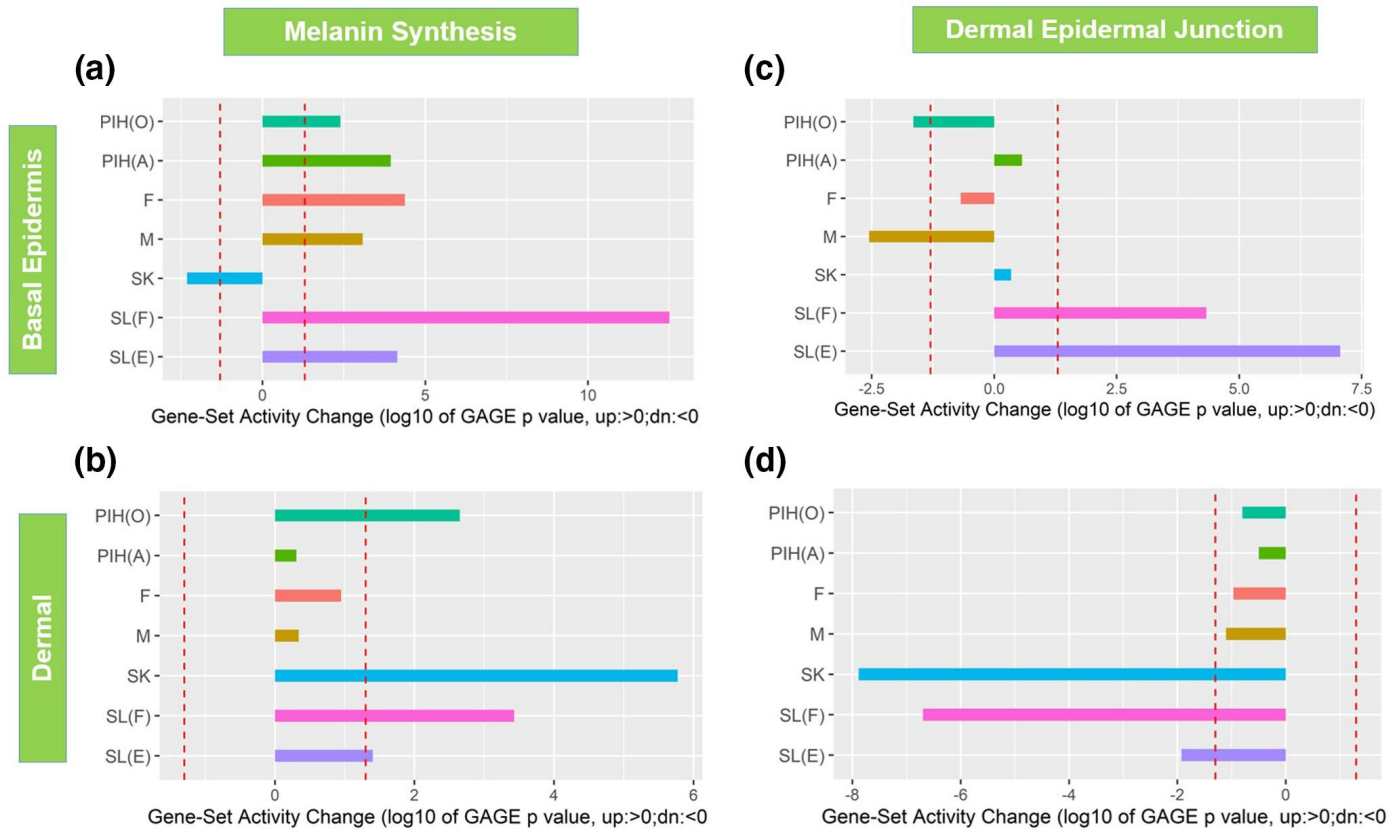
GAGE analysis on melanin synthesis (a and b) and dermal‐epidermal junction (c and d) related gene sets in basal epidermis (a and c) and dermal compartments (b and d). Red dash lines represent GAGE *p* = 0.05 thresholds. Log10 based GAGE *p*‐values are plotted with positive values representing upregulation and negative value representing downregulation of a gene set activity

Further transcriptome analysis was performed focussing on the commonly regulated biological themes or gene clusters across multiple spot types in melanogenesis. As expected, melanin synthesis pathway was upregulated in basal‐epidermis layer (enriched melanocytes) of all spot types except SK (Figure 5a). Interestingly, upregulation was also observed in the dermis for SL(F), SL(E), SK, and PIH(O) (Figure 5b), which were consistent with downregulation of DEJ related genes for SL(F), SL(E), SK in the dermis, and PIH(O) and Melasma in the basal‐epidermis layer (Figure 5c,d), suggesting impaired DEJ membrane leads to potential melanocyte infiltration into the dermis.

**FIGURE 6:**
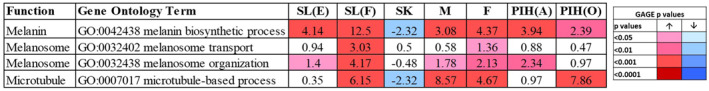
GAGE analysis in melanogenesis and microtubule activities for all spot types at basal epidermis layer based on GO terms. The numbers in the table are Log10(p) of GAGE *p*‐values; positive value indicate upregulation; negative values indicate downregulation. The colour legend indicates colour intensity with *p*‐value range

Melanosome transport and microtubule pathways were also upregulated in all spot types except for SK (Figure 6), indicating enhanced melanocyte dendricity and melanosome transportation in line with Pmel17 immunostaining observation (Figure 3).

### 4.4 Transcriptional evaluation of the epidermis in spot types

**FIGURE 7:**
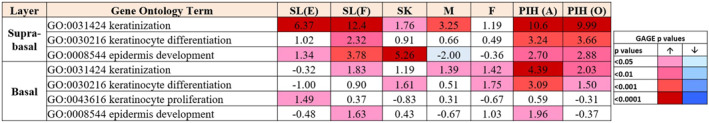
Key biological processes associated with epidermal development for all spot types at suprabasal epidermis and basal epidermis layers based on GO terms. The numbers in the table are Log10(p) of GAGE *p*‐values; positive value indicate upregulation; negative values indicate downregulation. The colour legend indicates colour intensity with *p*‐value range

**FIGURE 8:**
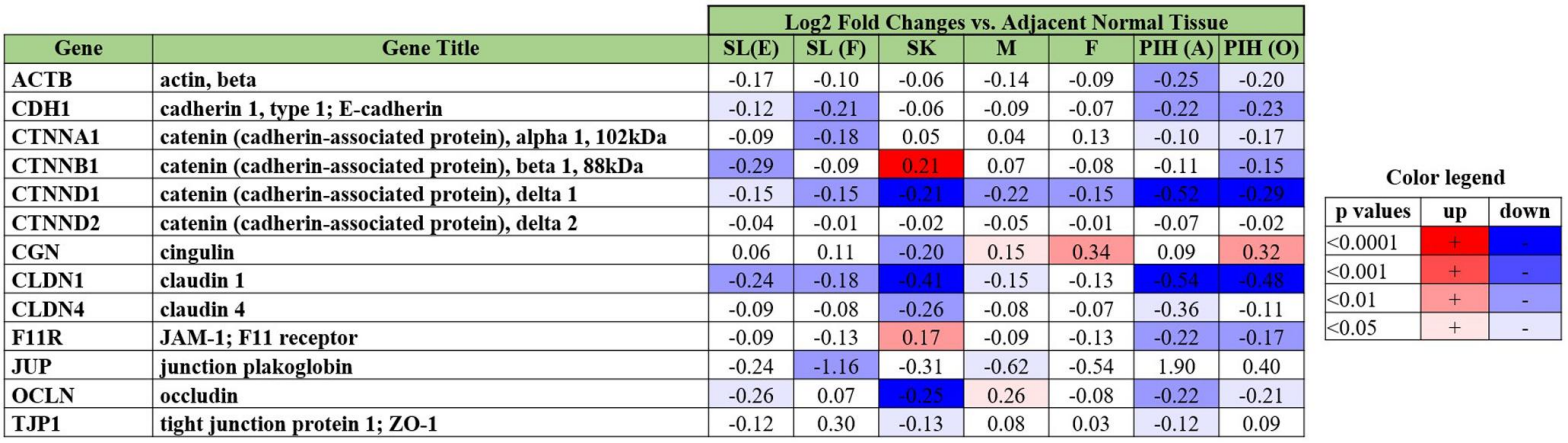
Regulation of key tight junction genes for suprabasal epidermis layer in all spot types. The numbers in the table are log2 fold changes versus adjacent normal tissue. The colour legend indicates colour intensity with *p*‐value range. Red indicates upregulation; blue indicates downregulation

**FIGURE 9:**
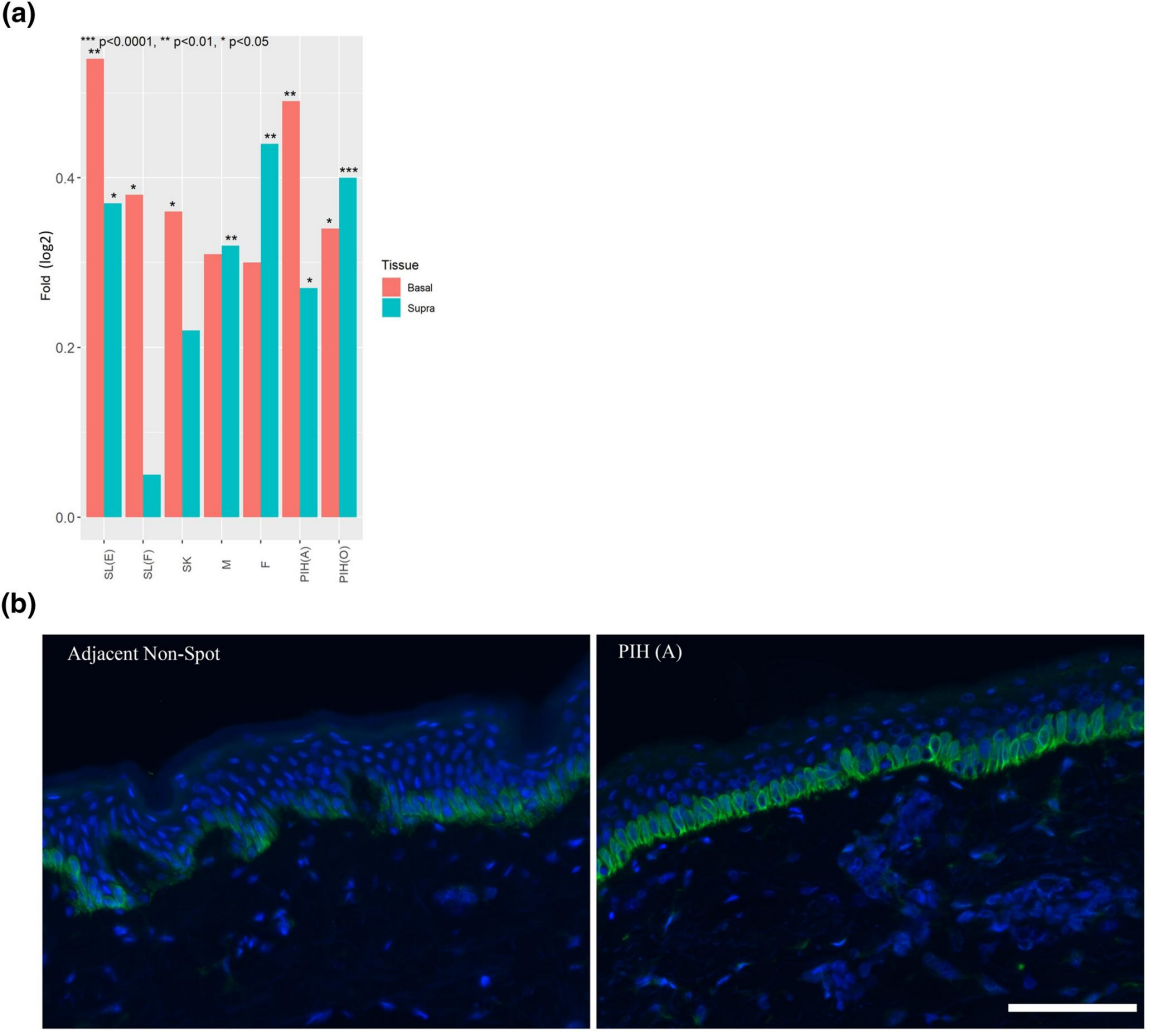
(a) Log2 fold changes of CDKN2A (a cellular biomarker of ageing and cell senescence) gene expression in both suprabasal epidermis and basal epidermis layers versus adjacent normal tissue for all spot types (b) Example images of CDKN2A (p16) protein immunostaining for PIH(a) spot tissue and non‐spot tissue. Scale bar = 100 microns

Further analysis identified interesting similarities across spot types in the epidermis beyond just pigmentation. First, key processes associated with epidermal structure and development were dramatically changed in all spot types (Figure 7). A general trend of upregulated keratinisation was observed in all spot types except Freckles. Keratinocyte differentiation and epidermal development were upregulated in SK and both PIHs in line with observed hyperplasia (Figure 1). Basal‐epidermis keratinocyte proliferation was significantly upregulated only in SL(E) which explains an elongated rete ridge. Secondly, downregulation of major tight junction genes was observed in multiple spot types (Figure 8). Specifically, claudin‐1 (CLDN1) was significantly down‐regulated in all spot types except Freckle, indicating potential impaired skin barrier function in majority of spots. Last, CDKN2A (p16), a cellular biomarker of cell senescence, was significantly upregulated in all spot types (Figure 9a). Images of CDKN2A immunostaining are shown (Figure 9b) as an example for PIH(A) and non‐spot tissue.

## 5 DISCUSSION

Various types of facial benign hyperpigmented spots represent global consumer concerns with physical and psychological impact.^22^, ^23^ However, the underlying molecular mechanisms for each type of spot are not fully elucidated. Among hyperpigmented spots, SL is extensively researched mechanistically, that is, showing upregulation of endothelin,^24^ stem cell factor,^25^ hepatocyte growth factor,^26^ potential spot initiation role of keratinocyte growth factor,^27^, ^28^ and involvement of heparanase to impair DEJ quality.^29^ Even including SL, limited transcriptomic and immuno‐histological studies were published on multiple spot types, and for the most studies, with tissue sampling from non‐facial sites and analysing full thickness biopsies.^3^, ^30^ Here, we reported comprehensive, skin compartmentalised transcriptional analysis with histological staining on multiple facial spots to characterise their underlying molecular biology and distinctive pathology.

It was previously reported that melanocyte density in photo‐exposed facial skin is higher than unexposed skin.^31^ Our study demonstrated most spot types exhibit similar melanocyte density in the basal epithelium (Figure 2d) which are somewhat surprising. Others reported SL on the back has higher melanocyte density than in adjacent non‐involved skin.^3^ This seems inconsistent with our observation, however, we normalized melanocyte count to undulated rete ridge whereas they normalized to stratum corneum, which could account for the discrepancy. In our study, uniquely, melanocyte density was increased in Melasma while slightly decreased in PIH(A). They are consistent with the literature for Melasma^32^ but is new and interesting finding for PIH(A), re‐enforcing the potential role of inflammation in pigmentation.

As expected, we observed increased expression of gene sets which regulate melanin synthesis (Figure 5a) across all spot types, indicating melanin synthesis activity, not melanocyte number, plays a prominent role in spot pigmentation although specific pattern varies by spot type (Figure S1). Since there is no uniform expression pattern of pigmentation genes for all spots, we presume the instigating causation is unique to each spot type. Also unique was SK, which demonstrated increased pigmentation (Figures 1 and 3) with minimal effect on the regulation of pigmentation genes. SK demonstrated a dramatic increase in epidermal and stratum corneum thickness and rete ridge undulation (Figure 2a–c), implying hyperpigmentation in SK results from the increased overall epidermal area as previously suggested.^33^

Furthermore, an increase in melanin production accompanied by increased melanocyte dendricity was confirmed by Pmel17 immunostaining for all spot types except SK (Figure 3). The finding was consistent with transcriptome analysis results (Figure 6) showing the upregulation of transcripts involved in melanosome transport and organization plus microtubule‐based processes occurring in all spot types, again except SK. These data provide evidence that activated infrastructure to transport melanosomes to keratinocytes including melanocyte dendricity also contribute to increasing visible pigmentation in spot areas. Enhanced melanocyte dendricity in spot area also implies more contact points to enable melanosome transfer to keratinocytes. When considering an intervention to reduce spot pigmentation, inhibition of melanocyte dendricity and/or melanosome transfer might be effective means to reduce total pigmentation in spots.

Upregulation of genes involved in melanin synthesis was also observed in the ‘dermis’ of both SLs, SK, and PIH(O) (Figure 5). The presence of melanocyte‐specific transcripts found in the dermis provides evidence that melanocytes (or dendrites) directly enter the dermal compartment. For these spots, DEJ related genes also exhibited significant upward and/or downward modulations at basal‐epidermis and/or dermal compartments. The modulations of DEJ genes suggest the DEJ maybe damaged or incomplete leading to porousness allowing; (a) higher levels of dermal paracrine factors to enter and affect the epidermal cells including stimulating keratinocyte differentiation/proliferation and melanogenesis, as well as (b) deposition and/or penetration of melanocytes due to impairment of the DEJ. It was previously reported that SL exhibits impaired DEJ due to heparanase.^29^ We also observed directional heparanase gene upregulation in suprabasal‐epidermis in multiple spots except SK (data not shown), suggesting it might be one of the central causes to induce DEJ damage. Of note, although we found the expression of melanin synthesis related genes in dermis compartment, the intensity was very low in the dermis compared with that expressed in the basal‐epidermis layer, suggesting that the amount of pigment residing in the dermis was limited, thus not observable by Pmel17 immunostaining (Figure 3). There are a few publications describing melanin presence detected in the dermis, that is, melanophages.^34^, ^35^ Further work in this area is needed as reported spot ‘permanence’^36^ may in fact be partially a result of dermal melanin deposition.

Further analysis was performed by focussing on the similarities across spot types beyond pigmentation pathways. First, in the suprabasal‐epidermis, we found the expression of genes which regulate keratinisation were significantly upregulated in all spot types except Freckles (Figure 7). These data are consistent with the observed increase in stratum corneum thickness (Figure 2c), suggesting upregulation of keratinisation leading to developing thicker stratum corneum in spot area as a common biological alteration. Interestingly, we observed upregulated keratinocyte proliferation in the basal‐epidermis layer ‘only for’ SL(E). It was previously reported^30^ SL(E) exhibits the upregulated keratinocyte proliferation and accounts for the elongated rete ridge,^37^ thus we expected the similar for SK and PIH, which revealed elongated rete ridge (PIH in trend). However, we also observed strong upregulation of epidermal development regulation and basal keratinocyte differentiation in SK and PIH, which correlate with longer rete ridge elaboration (Figure 2b). These transcriptome data could explain a different cause of elongated rete ridges for these spot types.

A significant difference between light versus dark skin complexion colouration is the status of melanosomes transferred into keratinocytes. Specifically, melanosomes in light skin are compartmentalised in membrane bound clusters within the cytosol of the recipient keratinocytes whereas melanosomes in dark skin are individually dispersed throughout the keratinocytes, and in both cases melanosomes are ultimately translocated apically over the nucleus.^38^, ^39^ Recently it was demonstrated that in Chinese skin the distribution pattern is intermediate between that in light versus dark skin, that is, approximately half of the incorporated melanosomes exist in clusters and half individually.^40^ The regulation of this differential distribution pattern is controlled by the keratinocyte cellular environment as opposed to the status of the imported melanosome.^41^, ^42^ In addition, keratinocytes from light skin can degrade melanosome more efficiently that from dark skin.^43^ Unknown is the distribution pattern and degradation rate of melanocytes in keratinocytes of hyperpigmented spots in general and if alterations of melanosome pattern and degradation rate in the spots of the different skin types correlates with the altered expression of genes which regulate keratinisation as described in the previous paragraph.

We also found multiple key genes that regulate the integrity of tight junctions between keratinocytes were generally down‐regulated in all spot types, except for Melasma and Freckles (Figure 8), specifically represented by a key tight‐junction gene, CLDN1. This data indicates potential impaired skin barrier function in spots due to downregulation of essential tight‐junction proteins connecting keratinocytes. Interestingly, this observation parallels consistently with observed DEJ modulation. Presumably, a weaker barrier function would allow external stressors to induce a paracrine response in keratinocytes that may stimulate melanin production in the melanocytes. Furthermore, as discussed, a damaged DEJ may allow crossover of various growth or proliferation factors into the epidermis potentially altering the rete ridge structure and keratinocyte proliferation/differentiation balance. In general, our data implies spot areas have lower expression of cell‐cell adhesive proteins, thus are leakier both at the surface and the DEJ.

Finally, CDKN2A, a senescence biomarker,^44^ was significantly up‐regulated in all spot types (Figure 9) indicating spot areas are biologically ‘older’ than non‐spot skin. Yoon et al. linked senescence to pigmentation by showing higher dermal senescence in solar lentigo resulted in lower stromal‐derived factor1 (SDF1) secretion, a pigmentation suppressor.^45^ We also observed directional down‐regulation of SDF1 in SL and statistically significant down‐regulation in PIH(A) and SK (data not shown), thus their finding of elevated senescence in SL is consistent with our observation of elevated CDKN2A. This further implies the impact of senescent cells seems to be a consistent theme for majority of spot types, thus may play a role in spot formation especially with ageing. Senescent cells are prone to creating an inflammatory micro‐environment, which decreases TGFβ release, resulting in higher pigmentation and lower repair processes such as autophagy.^46^ The inflammatory micro‐environment is also known to induce melanocyte dendricity,^47^ however, detail mechanism has not been elucidated. More research delineating the inflammatory pathways involved with pigmentation enhancement especially in spots is warranted.

We report herein a comprehensive study within Chinese females comparing the histological and transcriptional profiles of multiple spot types across three skin compartments collected via LCM. This provides a unique basis for high resolution analyses as well as creating customised intervention strategies either by targeting each type of hyperpigmented spot or addressing the biological similarities to effectively improve multiple spot types simultaneously. Future work to understand spot differences amongst different ethnicities is warranted to expand our holistic mechanistic knowledge of spots.

## CONFLICT OF INTERESTS

The authors declare they have no conflicts of interest.

## AUTHOR CONTRIBUTIONS

**T. Hakozaki:** Conceptualization; Project administration; Writing – original draft; Writing – review & editing. **B. Jarrold:** Formal analysis; Investigation; Methodology; Visualization; Writing – original draft; Writing – review & editing. **W. Zhao:** Conceptualization; Data curation; Formal analysis; Methodology; Software; Writing – original draft; Writing – review & editing. **T. Laughlin:** Investigation; Supervision; Writing – original draft; Writing – review & editing. **D. Whittenbarger:** Conceptualization; Data curation; Investigation; Methodology; Project administration; Supervision; Writing – original draft. **E. A. Jewell‐Motz:** Conceptualization; Funding acquisition; Project administration; Resources; Supervision; Writing – original draft. **R. E. Boissy:** Investigation; Supervision; Writing – original draft; Writing – review & editing.

## ETHICS STATEMENT

The study was approved by the RCRC Independent Review Board. Written informed consent was obtained from all particpants.

## Supporting information

Supporting Information S1

Supporting Information S2

## Data Availability

Research data are not shared.
