# Supplementary material for: Morphological and transcriptional evaluation of multiple facial cutaneous hyperpigmented spots
Source: Skin Health Dis. 2022 Feb 4;2(2):e96. doi: 10.1002/ski2.96 (PMC9168023; doi:10.1002/ski2.96)
Supplement: Supplementary file 1 — Supporting Information S1 [file SKI2-2-e96-s001.docx]

**SUPPLEMENTARY METHODS**

**LCM Processing to Isolate RNA from Three Skin Compartments**

For transcriptome analysis, biopsies were separated into three compartments (supra-basal epidermis, basal epidermis, and dermal) by LCM to enrich cell biological information. OCT embedded frozen tissue blocks were cut into 14µm sections, mounted on polyethylene napthalate slides (ThermoFisher, Waltham, MA) and kept at -80°C until use. Sections were immediately fixed in 95% ethanol for 1 minute then rinsed in deionized water. After rinse, the sections were stained with cresyl violet and eosin Y (Sigma Aldrich, St. Louis, MO) and rinsed with deionized water. The sections were dehydrated in ethanol, cleared in xylene, and air dried at room temperature (RT). The sections were subjected to LCM utilizing the PALM Microbeam system (Carl Zeiss Micro Imaging, München, Germany). Stated three compartments were captured for transcriptome analysis with the dermis defined as the tissue remaining following the removal of all epidermal appendages (hair follicles, sebaceous glands and sweat glands) and subcutaneous adipose tissue. Total RNA from LCM samples was isolated utilizing the Pico Pure RNA Isolation Kit (ThermoFisher) according to manufacturer’s recommendations. The quality and concentration of the isolated RNA was determined utilizing an Agilent 2100 Bioanalyzer and the RNA 6000 Pico Kit (Agilent Technologies, Santa Clara, CA) according to manufacturer’s recommendations.

**Histomorphometry**

Histomorphometry was conducted to observe structural alterations in different spot types compared to non-spot tissue. H&E staining was performed on 10µm sections from fresh frozen skin biopsies with the Shandon Rapid-Chrome Frozen Section Staining Kit (ThermoFisher) according to manufacturer’s recommendations. Multiple 20x bright field images of each biopsy were captured with an Olympus BX61 microscope, Cellsens DimensionTM software was utilized to stitch the images into one image encompassing the entire biopsy area. Image Pro Premier software (Media Cybernetics, Rockville, MD) was used to measure epidermal thickness, rete ridge content and stratum corneum thickness in each biopsy. Viable epidermis thickness was measured by tracing lines along both the DEJ and the epidermal granular layer then calculating the average distance between these lines across the entire epidermal length. Rete ridge content was measured taking the ratio of the DEJ length to that of the granular layer. Stratum corneum thickness was measured by tracing two separate lines along both the epidermal granular layer and the top of the stratum corneum then calculating the average distance between these lines across the entire epidermal length.

**Immunohistology of Pmel17, CDKN2A, and Microphthalmia Transcription Factor (MITF)**

10µm fresh frozen sections were fixed in ice cold acetone for 10 minutes at -20°C, washed in phosphate-buffered saline (PBS) and incubated for 1h at RT in 10% normal goat serum in PBS (Jackson ImmunoResearch, PA, U.S.A). Sections were incubated overnight at 4°C with either an anti-CDKN2A/p16INK4a (Abcam, MA, U.S.A, ab189034 1:100) or an anti-Pmel17 (Abcam, ab63297 1:15) antibody, washed in PBS, incubated with Alexa Fluor 488 conjugated goat anti-mouse or rabbit antibodies (Abcam, ab150117/ab150081 1:1000) for 1h at RT, washed in PBS and DAPI counterstained using NucBlue fixed cell stain Ready Probes reagent (ThermoFisher). For comparison, fluorescent images of spot and non-spot biopsies were captured with a Zeiss Observer.Z1 microscope (Carl Zeiss Microimaging, Germany) at equal gamma values, pixel range and exposure.

Identification/quantification of melanocytes in non-spot and spot biopsies (N=4 subjects for each spot type) was accomplished through MITF staining utilizing a MITF antibody (C5/D5 monoclonal, Sigma 284M-97) and a Histostain plus kit (ThermoFisher) with DAB as a chromogen according to manufacture recommendations. 10µm fresh frozen sections were fixed in ice-cold acetone for 10 minutes at -20°C, washed in PBS and endogenous peroxide activity was blocked with 0.3% hydrogen peroxide in PBS for 30 minutes at RT, washed with PBS, blocked with kit components, incubated with primary and secondary antibodies, washed, incubated with DAB for 4 minutes, counter stained with hematoxylin, dehydrated and cover slipped with Histomount (ThermoFisher). Multiple 20x bright field images of each biopsy were captured with an Olympus BX61 microscope, Cellsens Dimension^TM^ software was utilized to stitch the images into one image encompassing the entire biopsy area. Basal layer MITF positive cell nuclei were counted across the entire length of the DEJ of each biopsy. To account for differences in DEJ undulation between spot types and non-spot tissue all counts were normalized to 2mm of total DEJ length.

**Affymetrix GeneTitan mRNA target labeling, processing, and analysis**

For transcriptome analysis, purified RNA was converted to biotin-labeled complementary RNA copies using the Affymetrix HT 3’ IVT Express kit (Affymetrix, Santa Clara, CA) per the protocol provided and using a Beckman Biomek® FXp Laboratory Automation Workstation (Beckman, Indianapolis IN). 5 ng of total RNA was reverse-transcribed into cDNA using oligo-dT primers and reverse transcriptase followed by second strand synthesis using DNA polymerase I. Following purification, the cDNA library was used as a template for generating biotin-labeled cRNA copies using T7 RNA polymerase and biotinylated dUTP. Biotinylated cRNA was fragmented by limited alkaline hydrolysis and hybridized overnight to Affymetrix GeneTitan® U219 array plates using the Affymetrix GeneTitan® instrument and protocol provided. Following processing, chip images were converted to numeric data using the PLIER algorithm as executed in the Affymetrix Gene Chip Expression Console.
