# Supplementary material for: Morphological and transcriptional evaluation of multiple facial cutaneous hyperpigmented spots
Source: Skin Health Dis. 2022 Feb 4;2(2):e96. doi: 10.1002/ski2.96 (PMC9168023; doi:10.1002/ski2.96)
Supplement: Supplementary file 2 — Supporting Information S2 [file SKI2-2-e96-s002.docx]

**FIGURE S1**


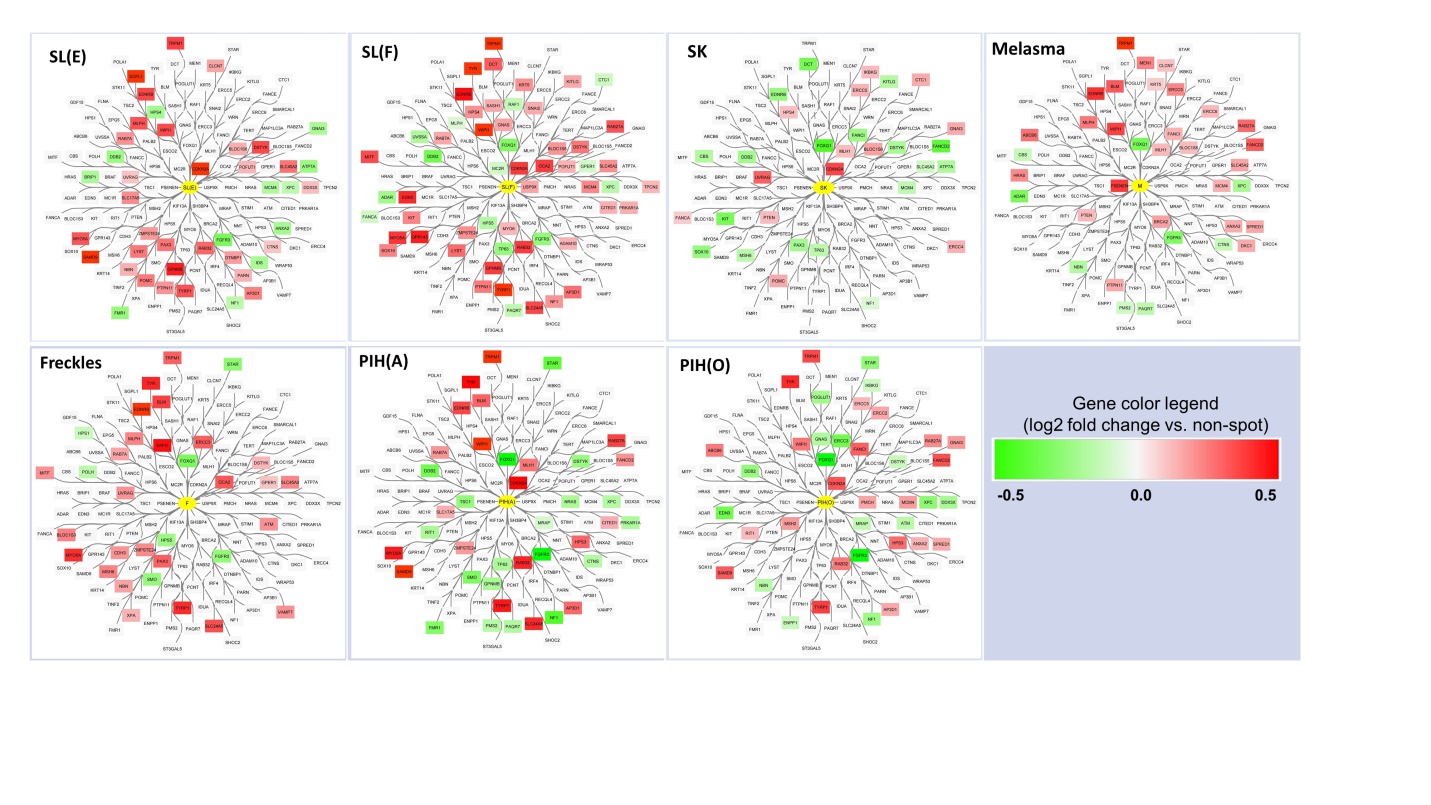


Figure S1. Cytoscape network graphs to visualize pigmentation gene expression differences at basal-epidermis layer for 7 spot types. 145 pigmentation related genes significantly regulated in at least one spot type are shown. In the graph, significantly regulated genes (p<0.05) are highlighted in red (upregulated) or green (downregulated).  The numbers of significantly regulated genes were; SL(E): 42, SL(F): 56, SK: 31, Melasma: 35, Freckles: 33, PIH(A): 39, PIH(O): 39.
